# Supplementary material for: Solvothermal synthesis of polyvinyl pyrrolidone encapsulated, amine-functionalized copper ferrite and its use as a magnetic resonance imaging contrast agent
Source: PLoS One. 2025 Feb 6;20(2):e0316221. doi: 10.1371/journal.pone.0316221 (PMC11801609; doi:10.1371/journal.pone.0316221)
Supplement: Attachment — Submitted filename: Answers_to_reviewers_questions_PONE_D_24_34021.docx [file pone.0316221.s002.docx]

**Reviewer #1:**

While the abstract mentions valuable insights and applications, it could further emphasize the broader implications of this research. How does this advancement contribute to the field of MRI or materials science in general?

1. Grammar and Word Choice:

- Line 29: There are some grammatical errors, such as "improved porperties in case of the of CuFe2O4-NH2 MW particles" which should be corrected to "improved properties in the case of the CuFe2O4-NH2 MW particles." The use of “of” twice in that sentence appears to be a typo.

**Thank you for your suggestion, it has been corrected.**

- Some sentences could be made more concise. For instance, the phrase "an innovative method was successfully applied and has yielded a novel particle with enhanced properties compared to traditional ferrite-based materials" could be streamlined. Consider rephrasing it to "This innovative method successfully produced nanoparticles with enhanced properties compared to traditional ferrite materials."

2. Materials:

- Please write the chemical formula correctly using subscripts.

**Thank you for your suggestion, it has been corrected.**

- Ensure you consistently use the same term (e.g., "ethanolamine" vs "ethanol amine") to avoid confusion.

**Thank you for your suggestion, it has been corrected.**

- Ensure consistency in the naming conventions used for compounds and solvents throughout the document. Example: Polyvinylpyrrolidone (PVP)

**Thank you for your suggestion, it has been corrected.**

- Line 116: “20 ml of ethanol amine was added” Please convert this to mmol.

**It was converted to mmol.**

- Line 116: The phrase "continuous continuous mixing" seems to be a typographical error; use "continuous stirring”

**Thank you for your suggestion, it has been corrected.**

3. Review of Results and Discussion

- The section describes the characterization of amine-functionalized magnetic nanoparticles synthesized through two different methods: reflux and microwave-assisted methods (CuFe2O4-NH2 Refl. and CuFe2O4-NH2 MW). Key aspects of the results are highlighted, including the crystallographic phases identified via X-ray diffraction (XRD) and specific phase compositions derived from Rietveld refinement.

**Thank you the suggestion, the follow section was added in the manuscript:**

*„Summarizing the XRD results, it can be concluded that the nanoparticles produced by microwave synthesis do not contain elemental copper. They contain two magnetizable phases, magnetite and copper ferrite, both of which are well suited as MRI contrast agents. The refluxed sample contains copper particles in addition to the magnetic phases mentioned above. The magnetite has crystallised on the surface of the copper, as copper is a good thermal conductor and a magnetic layer has formed on its surface, this raises the possibility of testing it in magnetic hyperthermia for cancer treatment in the future.”*

- The paragraph could benefit from clearer structuring. Consider breaking up long sentences for better readability. For instance, the sentence about the presence of copper peaks could be split to enhance clarity.

**The long sentences were broken.**

- Use subheadings where appropriate (e.g., “XRD Analysis” or “Particle Size Distribution”) to help readers navigate the section easily.

**The proposed subtitles have been added to the manuscript.**

- Line 206: "mesarured" which should be "measured,"

**Thank you for your suggestion, it has been corrected.**

- Provide a brief explanation or background on why the determination of these phases (CuFe2O4, magnetite, Cu) is significant for the application as MRI contrast agents. This context can help readers understand the relevance of the results.

The follows were added XRD results:

*“Using two different synthesis methods,* *CuFe_2_O_4_-NH_2_ Refl. and CuFe_2_O_4_-NH_2_ MW were prepared, these methods may result in differences in phase compositions. In order to confirmation, that the sample consisted of nanoparticles with the magnetic properties we wanted to produce, we needed to perform XRD measurements. XRD helps to detect possible contaminants (non-magnetic components) that may degrade the quality of the contrast agent, such as hematite or copper-oxides.”*

4. Ensure all statements regarding background information and previous research are properly cited.
5. Minor grammatical errors and sentence structure issues could be addressed for a more polished presentation. The overall tone is professional, but a thorough proofreading would enhance clarity.

**Thank you, we have made the linguistic corrections to the manuscript, and the English of the manuscript has been much improved.**

6. Incorporating an MTT assay in your study of copper ferrite nanoparticles would be a strong choice to assess the cytotoxicity, provided it is part of a broader strategy that may include additional assays for validation.

**AlmarBlue assay was assessed. The purpose of this was to show if Copper-ferrite would be an optimal platform for further development: 1) as a targeted therapeutic agent, 2) capable to provide contrast. We tested the toxicity on HEK293 cells for 24 hours, where we could detect a decreased cell number as the results of incorporating Copper atoms into the nanoparticle sample (CuFe_2_O_4_-NH_2_ Refl). These results are included in the supplemental material of this manuscript. However,**

**After a bolus injection into the tail vein of mice, a rapid dilution of twenty-times takes place. In vivo, acute effects or toxic effect were not observed after the injecting the particles into mice.**

**A more detailed toxicological study will be published on Copper-, Cobalt-, Zinc-ferrite and other particles, discussing their short- and long-term effects as well.** **The magnetite-coated copper nanoparticles will be tested later in hyperthermia cancer treatment in mice. This will also include toxicological tests, but these have not yet been carried out.**

7. A comparison with other published work must be performed to contextualize the findings and assess their significance concerning existing literature on similar nanoparticles." Especially with your published article [https://doi.org/10.3390/ijms242216203](https://doi.org/10.3390/ijms242216203) what is the impact of Cu compared to Zn

**Thanks for the suggestion, the results of zinc ferrite synthesis were compared with the results of copper ferrite synthesis. We have also explained why no pure spinel phase was obtained in the production of copper ferrite.**

*“In a previous publication, we reported on the preparation of zinc ferrite and its application as an MRI contrast agent. The synthesis methods were very similar to those used in the present work for the preparation of copper ferrite. In contrast to the present results, no other phases were found in the sample for zinc ferrite, only the spinel was formed during both preparation methods [https://doi.org/10.3390/ijms242216203]. In the case of copper(II) ions, reduction can occur, as ethylene glycol is an effective reducing agent in the conversion of Cu(II) ions to elemental copper nanoparticles. Ramyadewi et al. refluxed Cu(II) salt in ethylene glycol solution at 200 °C (as we did) and produced copper nanoparticles [https://doi.org/10.1016/j.matlet.2011.12.055].”*

8. The conclusions section should give the importance and usefulness of the research results

**We added the concluding part with the consideration that, our experiments have shown that the refluxing process can be used to successfully produce particles with a core-shell structure, with copper particles inside (which are good thermal conductors) and nanoparticles on the outside. This result raises the possibility that these core-shell particles could be used as magnetic hyperthermia treatment for cancers.**

**Reviewer #2:**

1. Add miller indices in xrd patterns

**Thank you, the suggestion, the Miller indices were added to the XRD patterns.**

2. Add error bar on statistical curve

**Error bars were added to the corresponding MRI figure (Fig. 11)**

3. Compare each obtained data with similar work

**The obtained results (XRD and VSM measurements) were compared with other works.**

4. The reason of using each precursor should be mentioned in exp. Section

*“In the course of our previous work, we have produced magnetic nanoparticles many times and found that ferrites can be efficiently produced starting from nitrate salts [https://doi.org/10.1038/s41598-024-54792-5] [https://doi.org/10.3390/ijms241713347].”*

5. Conclusion should be written more comprehensive

**Reviewer #3:**

The paper „Solvothermal synthesis of polyvinyl pyrrolidone encapsulated, amine-functionalized copper ferrite and its use as a Magnetic Resonance Imaging contrast agent“, written by Fatemeh Heydari et al., presents copper ferrite nanoparticles for contrast-enhanced MRI and a novel method of their preparation.

The authors present an improved synthesis method and compare nanoparticles prepared by a standard solvothermal synthesis and modified, microwave-assisted procedure. Particles prepared by both ways were compared by a row of physical measurements (magnetometry, X-ray diffraction, dynamic light scattering, relaxometry), and tested contrast enhanced MRI in vitro and in vivo. However, it is not clear, what is the main message: new particles for MR imaging, or the new procedure for preparation? Incorporation of copper into the nanoparticles might be interesting, but not from the point of view of MR imaging. Standard commercially available nanoparticles based on iron oxides have better performance (same or higher r2 relaxivity, substantially higher r1 relaxivity). If there is no special function of the copper in the nanoparticles (or of the particles themselves), there is no reason to use them instead of standard iron oxide nanoparticles. Therefore, I suppose, that the authors should emphasize the positive point of their work – the novel preparation method, thoroughly discuss the physical properties of nanoparticles prepared in a new way, discuss possible use of the method for preparation other nanoparticle types, and possibly think of submitting the paper to more technically oriented journal. I suppose that it should be rather rewritten than revised. I am sorry.

Specifically:

I appreciate a row of physical methods for characterization of nanoparticles; nevertheless, the paper suffers of many weak parts:

Language: I am not a native speaker, but I dare to say that the manuscript requires a thorough proofread. Many typos, mistakes or strange sentences strike even me. There are also typographic errors (physical quantities should be in italics, the authors should use subscript for numbers in chemical formulas, superscript for exponents etc.) throughout the text. Also, I think that there are wrong references to the images in the text

Point by point comments:

1) p. 3, l. 29: There should be plural of the verb “show”, I suppose. Typo in “properties”
2) p. 3, l. 30-32: Word order is strange.

**The word order has been changed, the sentence was rephrased.**

3) p. 4, l. 77-81: I have to remind, that NFS was extremely rare and since identifying the cause of NSF and adoption of relevant measures (such as avoiding application of linear Gd chelates to patients with renal failure), no case of NFS related to Gd contrast agents was reported. On the other hand, iron-oxide nanoparticles have much more frequent side effects (albeit not fatal) and there is no nanoparticle-based contrast agent currently approved for clinical use in Europe. Therefore, dreams of replacing Gd-based contrast agents by magnetic nanoparticles in near future are just wishful thinking (I do not oppose to study and explore magnetic nanoparticles, they are for sure very interesting and useful, but, please, do not defame Gd contrast agents, which are currently irreplaceable and I believe that they will be replaced by novel native MR sequences rather than by nanoparticles).

**Despite the reviewer’s suggestions, we still believe that other contrast materials besides Gd-chelates will be used in clinical practice in the near future. Of course, clinical trials and thorough examinations of such materials take time, but it is achievable. Economic aspects and significant advantages compared to Gd-materials must also be present. Additionally, the project should be backed by stakeholders and pharmaceutical companies. Our main goal is to describe these materials as thoroughly as possible, highlighting the potential pipeline towards the optimal candidate(s) for the future. Without wishful thinking, no change is achievable. Thank you very much for the comment.**

4) p. 4, l. 95-96: “Capping” agents or molecules is quite strange, I would recommend “coating”
5) p. 5, l. 117: Please, use RCF instead of RPM
6) p. 6, l. 133: “Techniques”, not “technics”

**Thank you for your comments, grammatical errors have been corrected in the manuscript based on the reviewers' suggestions.**

7) p. 8, l. 189-190: In vivo experiments are insufficiently described. How many mice the authors used? Which strain? Age? Sex? How was the suspension applied? Volume? Concentration? (Part of this information is in the Result section, but it should be here!)

**We have included the relevant information to the “Characterization Techniques” section.**

*“For the in vivo measurements, n=2 female, 10-week-old BalbC mice were used. The 0.2 mL of the 1 mg/mL contrast material was injected into the tail vein of the animals.”*

8) p. 8, l. 190-193: The described sequence (with a 90° excitation pulse and TR = 270 ms) has also quite strong T1-weighting, I suppose. Please, comment whether it may affect the results. Which organs were examined? How was the signal calculated/compared?

**The reason why the in vivo scans have a slight T1-weighting is that the sequence parameters were choosen to have fairly high liver signal on the native pre scans. That’s how the signal decrease could have been sufficiently high. As the r1 relaxivities are very low, we didn’t expect to have signal increase due to the T1 shortening effect even with the used paratmeter set. We have also added the following description to the manuscript:**

*“In InterView Fusion software (Mediso Ltd) 8 ROIs (liver, spleen, visceral fat tissue, renal pelvis, brain, muscle in the leg, cortex and medulla of the kidneys) were manually drawn on the pre scans of each mice. Images were normed to have same signal intensities in the muscle in order to have comperable signal intensity (SI) values. Relative signal changes were calculated in each ROI by the formula (SI_pre_ – SI_post_) / SI_pre_ to determine the biodistribution of the nanoparticles. The higher the measured signal intensity decrease the more the accumulated nanoparticles there.”*

9) p. 9: Figure 1 has no caption. Nevertheless, I think this figure is redundant.

**The function of figure one is to illustrate and summarize the whole study. We have corrected the mentioned figure and modified its caption. We added an explanation in the text. It has already become useful because it clearly illustrates the partial processes of our experimental work.**

10) p. 9, l. 214: The caption to Fig. 2 refers to A and B figures, however, there is only one graph in Fig. 2.

**Thank you for your comment, incomplete figure has been replaced.**

11) p. 10, Table 1: How many crystallites was evaluated to obtain average size (should be described in Methods)

**Two methods were used to determine the particle sizes, based on XRD results and TEM images. These results were compared.**

**The crystallite sizes in Table 10 were determined from XRD measurements, the method is described in the section Characterization techniques:**

*“Average crystallite size of the domains was calculated by the mean column length calibrated method using the full width at half maximum (FWHM) and the width of the Lorentzian component of the fitted profiles.”*

**The following addition has been made to the manuscript to clarify the measurement method based on the TEM recordings:**

*„**For the size analysis based on the TEM images, 100 particles were measured using a scalebar of pixel ratios from the TEM images. For the manual size determination, we used Image J [ImageJ: Image processing and analysis in Java WS Rasband - Astrophysics Source Code Library, 2012 - ui.adsabs.harvard.edu] [DOI: 10.17577/IJERTV4IS110211] https://doi.org/10.1016/j.compag.2008.02.007.”*

12) p. 10-11: What is the difference between Fig. 3 and 4? The caption is almost the same and it shows the same type of nanoparticles. Please explain.

**The two figures show images and elemental maps from different areas on the TEM grid of the CuFe_2_O_4_ Refl. sample. There were areas on the sample grid where only copper-ferrite nanoparticles were visible (Figure 3), but there were also areas where large particles of elemental copper were clearly visible (Figure 4). We wanted to show the extent to which morphological differences were observed in a single sample, namely CuFe_2_O_4_ Refl.**

**To make it easier to read and understand, we have added the following to the manuscript:**

*“During electron microscopy examination, we found sample-areas with significant morphological differences in the CuFe_2_O_4_-NH_2_ Refl. sample. In the HRTEM pictures,* *large nano- and microplates were found next to the above detailed copper ferrite nanoparticles also.”*

13) p.12, l. 268: “spectra”, not “spectrums”

**Thank you the suggestion, it was changed.**

14) p. 12, l. 272-273: The authors describe non-zero remanent magnetization of the nanoparticles and conclude that “the nano powder is paramagnetic in nature”. Existing remanen magnetization contradicts paramagnetism!

**Thanks for your comment it has been corrected.**

15) p. 12, l. 270-279: There is a substantial difference in saturation magnetization between the two nanoparticle types (Ms of CuFe2O4-NH2 Refl is three times higher than Ms of CuFe2O4-NH2 MW), although they are chemically identical. I miss there an explanation or at least a hypothesis. This is crucial for the nanoparticle performance.

**The difference between the Ms values were reported in the manuscript:**

*“**A significant difference is observed in the saturation magnetization values of the two samples. The Ms value of the CuFe_2_O_4_-NH_2_ Refl. sample was several times higher than that of the CuFe_2_O_4_-NH_2_ MW sample. XRD analysis also showed that the CuFe_2_O_4_-NH_2_ Refl. sample contained a much higher amount of magnetite (32.6 wt%) than its microwave synthesized counterpart (23.2 wt%) (Table 1.). Furthermore, considering that, the bulk phase magnetite has a Ms value of 90-92 emu/g, whereas the copper ferrite array phase has a lower Ms value (49-55 emu/g), it can be assumed that the quantitative relationship between the two different magnetic components also contributes to the different Ms values for the two samples produced. [DOI: 10.2174/156652312800099616][* [*https://doi.org/10.1063/1.372662*](https://doi.org/10.1063/1.372662)*]. The two samples show a significant difference in the size of the copper ferrite and magnetite particles (Table 1). As we know that, the saturation magnetization value decreases with decreasing particle size due to the spin disorder at the surface of the nanoparticles, which leads to the significant change of the Ms values at very small dimensions [[Costica Caizer](https://link.springer.com/referenceworkentry/10.1007/978-3-319-15338-4_24#auth-Costica-Caizer), Nanoparticle Size Effect on Some Magnetic Properties Reference work entry pp 475–519, Handbook of Nanoparticles, DOI 10.1007/978-3-319-15338-4_24]. This explains the large difference in saturation magnetization values measured for two copper ferrite samples. The average sizes of the magnetic nanoparticles (magnetite and copper ferrite) are larger for the CuFe_2_O_4_-NH_2_ Refl. sample, which leads to a higher Ms value. A similar trend has been reported in the literature, where 37.98 emu/g was measured with size ranging from 20 to 40 nm for CuFe_2_O_4_, 39 emu/g at an average size of 50 nm and 30 emu/g Ms in the 10-20 nm size range [DOI: 10.1080/15533174.2012.680090] [Farghali, A.A.; Khedr, M.H.; Khalek, A.A. J. Mater. Process. Tech. 2007, 181, 81] [Kalai Selvan, R.; Augustin, C.O.; Sanjeeviraja, C.; Prabhakaran, D. Solid State Commun. 2006, 137, 512.].”*

16) p. 12, l. 278: The statement “The PVP-stabilized ferrites showed high stability in their aqueous colloids“ might be correct, however, experiments proving it are described later in the paper, therefore, save it for following paragraphs. Does the colloid redisperse itself when it is magnetized (see Fig. 7C)?

**Thank you for your comment, that sentence has been moved to the DLS chapter. The Nd magnet can be used to collect the nanoparticles, but after removing the magnet, the particles are into colloidal form by shaking** .

17) p. 12, l. 282-283: The sentence is incomprehensible to me. How can you get concentration 1 mg/mL by dispersing of 100 mg in 5 mL???

**The wording may have been ambiguous thus it has been corrected.**

*„The concentration of copper-ferrite nanoparticles stabilized in PVP support was 50 mg/g. Accordingly, 100 mg of PVP-stabilized copper ferrite contains 5 mg of CuFe_2_O_4_ nanoparticles. If 100 mg of PVP-supported copper ferrite sample was dispersed in 5 ml of distilled water, the concentration of the magnetic component is 1 mg/ml.”*

18) p. 13, l. 285-294: I am puzzled by the terms of “rectangularity”, however, I am not familiar with AFM, so it may be just lack of knowledge on my side.

**Our main goal was describing the particles as good as possible. Built-in analysis software are of use in this case. The built-in software of the Cypher-S AFM, IGOR has analysis functions not only to measure the size-distribution of nano-sized objects, but also to determine the circularity (e.g. in case of bacteria) or the rectangularity (e.g. in case of nanoparticles). By the definition of Asylum Research (the manufacturer), “The ratio of the area of the particle and the area of a rectangle that has the same 1st and 2nd moments of the particle. The closer a particle is to a rectange the closer the value to 1 is.”**

19) p. 13, l. 301-306: The colloid stability was really measured within three hours only? It is insufficient for in vivo applications!

**Copper ferrite nanoparticles were embedded in a PVP support and dried into a solid form because they can be used for a very long time (no coagulation). It must be dispersed in distilled water immediately before the MRI examination and then injected into the animal. We performed a three-hour DLS measurement to see if the injection is stable for at least that long after preparation. We did not perform longer measurements because the DLS measurement in distilled water does not provide information about what happens with the size distribution when in the living organism (blood stream and tissues). We must not forget that blood is a complex colloidal system that contains many particles (red blood cells, white blood cells, etc.) and different electrolytes, and there is a continuous flow at a given temperature. Thus, the DLS measurement is not suitable for detecting how the particle size changes in the bloodstream.**

20) p. 14, l. 311-321: This part are not results. Explanation of relaxation times in MRI and their role in clinical practice belongs rather to the Introduction. Following sentence belongs to the Methods.

**Thank you for the suggestion. The referred section is moved to the Mateirals & Methods.**

21) p. 14, l. 328: Reference to Fig. 9 should be probably Fig. 10.
22) p. 15, l. 336: Fig. 11 instead of 10?
23) p. 15, l. 339: Fig. 11 instead of 10?

**Thank you, the figure numbers were corrected.**

24) p. 15, l. 345-350: The authors conclude that “relaxivities were in the same range as Feraheme and Endorem”, I would expect somewhat more precise comparison. It requires some calculation, as relaxivities are usually presented in [s-1/mM]. Recalculated r2 for CuFe2O4-NH2 Refl is 58.8 s-1/mM, and for CuFe2O4-NH2 MW 95.9 s-1/mM (the coating was neglected – by the way, it is not clear, whether the measurements were performed on coated particles), while Endorem has r2 = 158 s-1/mM and Ferahem 83 s-1/mM (both at lower field 1.5 T, so the values will be even higher at 3 T), see e.g., <http://dx.doi.org/10.1055/s-0033-1335438>
    Also, I would expect substantially higher relaxivity in the case of CuFe2O4-NH2 Refl, which has three times higher saturation magnetization and much steeper magnetization curve than CuFe2O4-NH2 MW. Can you comment on this?

**Thank you for the comment. All measurements were performed on PVP-coated nanoparticles – Fig 11 was corrected accordingly. Of course, relaxivity values are depending on the sequence applied, temperature, magnetic field strength and medium applied (where the particles are suspended). Moreover, the colloidal stability and molecular structure is also highly influential on this matter. The colloidal stability of the CuFe_2_O_4_-NH_2_ MW particles, based on the DLS measurements was superior to the CuFe_2_O_4_-NH_2_ Refl particles. In a different perspective, the “poorer” colloidal performance influenced the saturation magnetization and magnetization curve.**

*“Comparing the CuFe_2_O_4_-NH_2_ samples (manufactured with the conventional, lengthy synthesis and the microwave-initiated synthesis) to standard superparamagnetic iron oxide (SPIO) nanoparticles, to which they belong according to their hydrodynamic size (127 nm and 242 nm), some different characteristics had been found. The r1/r2 ratios of our samples were an order of magnitude lower, as Endorem® (with size of 80-150 nm) has r1/r2 ratio 0.044  and Resovist® (size of 62 nm) has r1/r2 ratio 0.032 [Comparison of Magnetic Properties of MRI Contrast Media Solutions at Different Magnetic Field Strengths (M. Rohrer et al., Invest Radiol 2005;40: 715-724)]. It suggests, that our ferite solution is almost only a T2 contrast agent and has an undetectable T1 effect (65). On the other hand, Resovist has 1.5-times as high r2 relaxivity at 3 Tesla as Endorem, the same relationship, that we found between the size and the r2 relaxivity of our samples.”*

25) p. 15, l. 351: I miss an information about the safety of the nanoparticles prior to in vivo application to experimental animals. Did authors perform cytotoxicity tests? Did they test the long-term colloidal stability? Is the PVP coating stable? PVP itself is soluble in water!

**Cytotoxicity test were conducted, where ferrite nanoparticles did not exhibit however a more thorough investigation regarding long-term effects (based on pathological examination) of ferrite, magnetite and maghemite nanoparticles will be summarized in upcoming manuscript as well. Ferrite nanoparticles exhibited similar results as distilled water (MQ) injected intravenously (considering the standard deviation as well). The new synthesis resulted in a lower suspected long-term toxicity, based on the short-term results, on cells. The toxicity of any sample examined decreases over the dilution. In vivo, after a bolus injection into the tail vein of mice, a rapid dilution of twenty-times takes place. This dilution factor in humans is usually larger. The results are included in the supplemental information.**

**Due to excellent biocompatibility and stabilizing effect of the polyvinylpyrrolidone, it is used for the synthesis and stabilization of MRI contrast agents (such as magnetite) [DOI:10.3144/expresspolymlett.2010.42]. Molecular Dinamics (MD) calculations were confirmed, that the PVP polymer interacts physically with magnetite cluster via van der Waals with an average binding strength of −9.2 kcal mol^−1^ [https://doi.org/10.1039/D0EN00907E]. Similar molecular dynamics calculations for copper ferrite particles have not been found, but it is assumed that a similar level of interaction occurs between PVP and ferrite particles. Thus, the PVP coating on surface of the magnetic nanoparticles shows sufficient stability to prevent aggregation of particles; dissolution of PVP from the particle surface is not expected.**

26) p. 16, l. 354-358: The authors state that “An immediate uptake was observed in the liver and in the spleen. No other organ accumulated the sample according to our T2*-weighted gradient echo scans.“ However, on line 357, they write that “…liver, kidney, vena cava and spleen uptake are clearly visualized“. Please, correct

**Thank you for the comment. The figure has been corrected. Liver and kidney has been marked.**

27) Formal omissions:

Funding should include initials of the authors who received each award (required by the publisher)
Missing explanation, why the data are not available („Stating ‘data available on request from the author’ is not sufficient. If your data are only available upon request, select ‘No’ for the first question and explain your exceptional situation in the text box.“)

**The funding information was corrected, information was added with the initials of the authors. Data will be shared in the supplemental information section.**

**Reviewer #4:**

1. Characterization technics is wrongly written. please check the spellings. In introduction section, I suggest authors to add some more information about substitution. In the introduction, there is a lack of application; at the same time, some recent technological developments are missing. Strongly mention the novelty of the paper and why do authors choose this method? Also, please highlight what's new in yours.

**The following has been added to the introduction of the manuscript.**

*“As detailed above for the different synthesis methods, we have seen that significant attention is paid to the various solvothermal processes, within which microwave synthesis is often used. In our work, we have compared the solvothermal synthesis methods applicable to the synthesis of amine-functionalized copper ferrite, namely microwave irradiation and reflux boiling. The same precursors and quantitative ratios were used in both synthesis methods. Magnetic naor particles produced during synthesis were compared in terms of size, morphology, magnetization properties, colloidal instability and phase composition. We also tested the magnetic particles as MRI contrast agents. The results obtained were used to compare the efficiency of the two synthesis methods in the production of copper ferrite.”*

**Another novelty of this work is the “instant” formulation of amine functionalized copper ferrite and magnetite nanoparticles using a water-soluble, biocompatible drug delivery agent, namely polyvinylpyrrolidone, which are used for stabilization of vaccines. The magnetic nanoparticles were embedded in a polyvinylpyrrolidone matrix, resulting in a solid formulation that does not aggregate and retains its quality for a very long time in its dried state. Dissolved in water immediately before injection, the magnetic nanoparticles which are stearically stabilized with** **polyvinylpyrrolidone are easily dispersed and form a stable colloid for hours.**

2. The authors must highlight the limitations of the proposed method and discuss about future improvements/works.

**To explain future plans, the following text has been added to the manuscript.**

*“Summarizing the XRD results, it can be concluded that the nanoparticles produced by microwave synthesis do not contain elemental copper. They contain two magnetizable phases, magnetite and copper ferrite, both of which are well suited as MRI contrast agents. The refluxed sample contains copper particles in addition to the magnetic phases mentioned above. The magnetite has crystallized on the surface of the copper, as copper is a good thermal conductor and a magnetic layer has formed on its surface, this raises the possibility of testing it in magnetic hyperthermia for cancer treatment in the future.”*

3. The XRD patterns should be refined using the Rietveld method by using relevant software. The relevance factors (chi^2, RB, etc) of XRD patterns fitting should be added. The possible reasons for the variations (increase or decrease) in lattice parameters and crystallites size should

be provided in the manuscript.

*„**Identification of crystalline components was done by Search/Match, on ICDD PDF2 (2005) database in the Bruker DiffracPlus EVA software. Quantitative composition was calculated with Rietveld refinement in Bruker TOPAS4 by fundamental parameters approach (FPA) and the generated instrumental profile was validated on SRM640a Si standard. Crystallographic information for the identified phases was taken from ICSD and AMCSD databases [R. A. Young, The Rietveld Method, Oxford University Press, 1995]*

*[L.B. McCusker, R.B. Von Dreele, D.E. Cox, D. Louër, P. Scardi, Rietveld refinement guidelines, J. Appl. Crystallogr. 32 (1999) 36–50.* [*https://doi.org/10.1107/S0021889898009856*](https://doi.org/10.1107/S0021889898009856)*] [R. E. Dinnebier, A. Leineweber, J. S. O. Evans, Rietveld Refinement: Practical powder diffraction pattern analysis using TOPAS, De Gruyter, 2018]”*

**The relevant factors of the XRD patterns fittings are the follows:**

**- CuFe_2_O_4_ MW: chi2  : 3.93   GOF : 0.53 DW  : 0.05**

**- CuFe_2_O_4_ NH2 12h: chi2 : 3.86   GOF : 0.54 DW  : 0.04**

**GOF = goodness of fit, closer ot 0, better the fit (R. E. Dinnebier, A. Leineweber, J. S. O.)**

**DW = Drubin and Wattson statistics, closer ot 0, better the fit (Durbin & Watson, 1971)**

**The possible reasons for the variations (increase or decrease) in lattice parameters and crystallites size the follow:**

*„**In the case of CuFe_2_O_4_-NH_2_ Refl. samples the synthesis time was 12 hours at 200 °C by which the crystal growth is facilitated, while the CuFe_2_O_4_-NH_2_ MW sample was only 5 minutes exposed to the same conditions. Thus, larger crystals were developed by the longer exposure time.”*

**The paper does not discuss lattice parameters, however if it would, than the variations should refer to the lattice strain of the given samples.**

4. The depth of technical discussions needs to go much further. Vague explanation is provided

**Thank you for your suggestion, we have explained our results and experimental techniques in more depth and extended our evaluation.**

5. Authors should compare their results with the recently published articles (2022/2023) in this field.

**Our measurement results were compared with results from international literature**.

6. There is total ignorance of magnetic study by the fitting of some equation. You can see the article <https://doi.org/10.1002/pssb.201800676> and <https://doi.org/10.1007/s00339-019-2615-8>

**The above passage can be interpreted that the Langevin-fit was applied to the ferromagnetic CuFe_2_O_4_-NH_2_ Refl. sample on fig. 7 A, as the reviewer noted, this would be completely wrong. In fact the Langevin equation was used only to the superparamagnetic CuFe_2_O_4_-NH_2_ MW sample on fig7a. We change the misleading part of the text:**

*„These suggest that the nano powder is ferromagnetic (Fig. 7 A). The magnetic saturation (Ms) was estimated from fig.7a as 44 emu/g. In case of CuFe_2_O_4_-NH_2_* *MW sample the magnetization curve could not be brought to saturation even with the maximum applied magnetic field strength and hysteresis was not observed as well. This refers to the superparamagnetic nature of the sample, so the saturation magnetization was calculated using the fit of the Langevin function. [ref:* [*Journal of Nanomaterials*](https://onlinelibrary.wiley.com/journal/9182) *[Volume 2019, Issue 1](https://onlinelibrary.wiley.com/toc/9182/2019) 6409210]”*

**Reviewer #5:**

Solvothermal synthesis of polyvinyl pyrrolidone encapsulated, amine-functionalized
copper ferrite and its use as a Magnetic Resonance Imaging contrast agent
The manuscript reports the preparations of polyvinyl pyrrolidone /amine-functionalized copper

ferrite NPs for MR Imaging application.

In general, the manuscript falls well within the scope of Plos One and has its merits to support its consideration of publication from a standpoint of functional materials design and preparations. The following issues please be addressed before the recommendation of publication.

Comments 1.

The abstract could be written with literature values.

**Data have been added to the abstract.**

Comments 2

The paper failed to explain the novelty of the prepared MRI contrast agent with other reported agents. The author must rewrite the introduction section to properly explain the novelty of synthesis method.

**The manuscript has been supplemented by the following:**

“As detailed above for the different synthesis methods, we have seen that significant attention is paid to the various solvothermal processes, within which microwave synthesis is often used. In our work, we have compared the solvothermal synthesis methods applicable to the synthesis of amine-functionalized copper ferrite, namely microwave irradiation and reflux boiling. The same precursors and quantitative ratios were used in both synthesis methods. Magnetic nanoparticles produced during synthesis were compared in terms of size, morphology, magnetization properties, colloidal instability and phase composition. We also tested the magnetic particles as MRI contrast agents. The results obtained were used to compare the efficiency of the two synthesis methods in the production of copper ferrite.”

**Another novelty of this work is the “instant” formulation of amine functionalized copper ferrite and magnetite nanoparticles using a water-soluble, biocompatible drug delivery agent, namely polyvinylpyrrolidone, which are used for stabilization of vaccines. The magnetic nanoparticles were embedded in a polyvinylpyrrolidone matrix, resulting in a solid formulation that does not aggregate and retains its quality for a very long time in its dried state. Dissolved in water immediately before injection, the magnetic nanoparticles which are stearically stabilized with polyvinylpyrrolidone are easily dispersed and form a stable colloid for hours.**

In the Introduction, section, lines 65-66, page 3, There is no citation for the sentence

lines 65-66, the sentence was not cited.

**The reference was added for the above-mentioned sentences.**

Comments 3

The properties in terms of physical and biological activity of the fabricated NPs should be compared with other commercially reported contrast agent previously in the main text,. Otherwise, it is difficult to claim the superiority of this product.

**Thank you for your insightful comment. We acknowledge the challenge in comparing different materials, particularly nanoparticles with non-nanoparticulate agents. Our primary focus is on the in vitro and in vivo contrast capabilities of the fabricated nanoparticles, which we evaluate based on relaxivity data, stability, and in vivo performance.**

**The purpose of this study is to compare two identical contrast materials, emphasizing the “superior” performance of one in terms of economic advantages for manufacturing and stability. While a comparison with Gd-based materials is included to provide context within the field of MRI imaging, our main objective is to highlight the specific benefits of our nanoparticles**

Comments 4

Please include the model no, brand name, and origin of instruments used in this work. For example, general information about the Bruker D8 equipment, Bruker Vertex 70 equipment, Weiss-type electromagnet, etc was not given in the paper.

**The requested data have been added to the manuscript.**

Comments 5

Please follow the same notation in mentioning units like ml, minutes, hours, etc....for example, if authors used hr, hrs, hour, and hours to mention time please check all units and follow a specific notation of short forms for the same.

The author must write properly the subscript in the manuscript. For instance, CuFe_2_O_4_-NH_2_

**We have standardised the unit notations.**

Comments 6

The influence of cross-linking time and mass ratio of crosslinker on the mechanical performances and also the biological behaviors of the hydrogel can be expanded in a discussion.

**We thank the reviewer for pointing out an important aspect of the research. All molecular measurements are included in the revised version of the manuscript, including FTIR, XRD and VSM measurements.**

Comments 7

In Figure 1, rewrite the caption. It does not clearly explain the figure 1. The abbreviation of

PVP and MEA could be included in the material sections. Figure 1 could be named as Scheme 1

**We have changed the caption on the figure and rewritten the experimental section so that the figure helps you to quickly review our work.**

I suggest authors to add some molecular indexes analysis to increase the publication value of the paper.

**We thank the reviewer for the insightful suggestion. We are considering doing so, however the amount of information in this recent manuscript might be overwhelming for the readers.**

**Reviewer #6:** Minor revision is required.

English need to be written in standard form. The editing by a native speaker or professional language editing service must be utilized to correct the overall english writing.

**Thank you for your comments, we have proofread the manuscript in English.**
